# Neuroretinal Cell Culture Model as a Tool for the Development of New Therapeutic Approaches for Oxidative Stress-Induced Ocular Diseases, with a Focus on Glaucoma

**DOI:** 10.3390/cells13090775

**Published:** 2024-05-01

**Authors:** Kristian Nzogang Fomo, Natarajan Perumal, Caroline Manicam, Norbert Pfeiffer, Franz H. Grus

**Affiliations:** Experimental and Translational Ophthalmology, Department of Ophthalmology, University Medical Centre of the Johannes Gutenberg University Mainz, 55131 Mainz, Germany; jnzogang@uni-mainz.de (K.N.F.); nperumal@eye-research.org (N.P.); caroline.manicam@unimedizin-mainz.de (C.M.); norbert.pfeiffer@unimedizin-mainz.de (N.P.)

**Keywords:** glaucoma, neurodegeneration, oxidative stress, proteomics, retina

## Abstract

Glaucoma is a heterogeneous group of optic neuropathies characterized by a progressive degeneration of the retinal ganglion cells (RGCs), leading to irreversible vision loss. Nowadays, the traditional therapeutic approach to glaucoma consists of lowering the intraocular pressure (IOP), which does not address the neurodegenerative features of the disease. Besides animal models of glaucoma, there is a considerable need for in vitro experimental models to propose new therapeutic strategies for this ocular disease. In this study, we elucidated the pathological mechanisms leading to neuroretinal R28 cell death after exposure to glutamate and hydrogen peroxide (H_2_O_2_) in order to develop new therapeutic approaches for oxidative stress-induced retinal diseases, including glaucoma. We were able to show that glutamate and H_2_O_2_ can induce a decrease in R28 cell viability in a concentration-dependent manner. A cell viability of about 42% was found after exposure to 3 mM of glutamate and about 56% after exposure to 100 µM of H_2_O_2_ (*n* = 4). Label-free quantitative mass spectrometry analysis revealed differential alterations of 193 and 311 proteins in R28 cells exposed to 3 mM of glutamate and 100 µM of H_2_O_2_, respectively (FDR < 1%; *p* < 0.05). Bioinformatics analysis indicated that the protein changes were associated with the dysregulation of signaling pathways, which was similar to those observed in glaucoma. Thus, the proteomic alteration induced by glutamate was associated with the inhibition of the PI3K/AKT signaling pathway. On the other hand, H_2_O_2_-induced toxicity in R28 cells was linked to the activation of apoptosis signaling and the inhibition of the mTOR and ERK/MAPK signaling pathways. Furthermore, the data show a similarity in the inhibition of the EIF2 and AMPK signaling pathways and the activation of the sumoylation and WNT/β-catenin signaling pathways in both groups. Our findings suggest that the exposure of R28 cells to glutamate and H_2_O_2_ could induce glaucoma-like neurodegenerative features and potentially provide a suitable tool for the development of new therapeutic strategies for retinal diseases.

## 1. Introduction

Retinal diseases are a significant health concern as they can cause irreversible blindness by affecting various parts of the retina [1]. These diseases are commonly caused by inflammation and oxidative stress or other pathological conditions that can lead to metabolic disorders [2,3], resulting in the death of retinal cells or adjacent supporting tissues. There are several types of retinal diseases, including age-related macular neurodegeneration (AMD), diabetic retinopathy (DR) and glaucoma.

Glaucoma is a group of eye diseases characterized by the progressive degeneration of retinal ganglion cells (RGCs) and an irreversible loss of vision [4]. Glaucoma affects more than 70 million people worldwide, with approximately 10% suffering from bilateral blindness. This makes it the leading cause of irreversible blindness in the world [4]. In addition, approximately 4% to 7% of individuals over the age of 40 have a prevalence of ocular hypertension (OHT), but only about 1% of the risk group develops glaucoma per year [5]. With a prevalence of 3.54% in people aged 40 to 80 years, the number of glaucoma patients worldwide was estimated to be 64.3 million in 2013, and the projection for 2040 is 111.8 million people [6]. To date, the exact biological mechanism of glaucoma is not fully understood, but besides increased intraocular pressure (IOP), which is the main risk factor, other concomitant pathological factors affecting the eye have been proposed, such as increased glutamate levels [7], alterations in nitric oxide (NO) metabolism [8], vascular alterations [9], autoimmunity [10] and oxidative damage caused by reactive oxygen species (ROS) [11,12]. Nowadays, the management of the IOP still represents the main therapy for glaucoma, but it is not sufficient to fully prevent disease progression. Therefore, the development of new perspectives for the treatment of glaucoma represents an attractive field for the future. To this end, the development of models such as the experimental animal model (EAM) of glaucoma has greatly contributed to the understanding of the pathological mechanisms of glaucoma [13] and to the development of new therapeutic approaches [14]. Nevertheless, there is a recognized need for in vitro models of glaucoma in order to allow broad screening of potential glaucoma drug candidates and to reduce the quantity of animals used for experimental purposes.

Glutamate is an essential amino acid that acts as a major excitatory neurotransmitter in the central nervous system and the retina [15]. Therefore, glutaminergic neurons represent the main excitatory system of the brain, playing an important role in the regulation of neurological functions in mammals [15]. However, the effects of glutamate in the brain and retina are exerted through several receptors, namely the metabotropic glutamate receptors (mGluR) and the ionotropic glutamate receptors (iGluR), which include the N-methyl-D-aspartic acid (NMDA), α-amino-3-hydroxy-5-methylisoxazole-4-propionate (AMPA) and kainic acid (KA) receptors [16]. In general, the stimulation of ionotropic receptors by glutamate leads to Ca^2+^ influx and the depolarization of the postsynaptic membrane, thereby promoting excitatory neurotransmission [17]. On the contrary, excessive stimulation of ionotropic receptors by glutamate might trigger an ionic imbalance, leading to cell death through a process called excitotoxicity [18,19]. Moreover, glutamate toxicity may also result in an imbalance of the redox system due to a blockage of the cystine/glutamate antiporter, which inhibits the cystine uptake necessary for the production of the intracellular reducing agent glutathione. This in turn leads to an increase in ROS production, resulting in cell dysfunction and apoptosis [20].

The R28 cell line is a retinal precursor cell line that was established from a postnatal day 6 rat retina culture immortalized with the adenovirus 12S E1A gene (NP-040507) in a replication incompetent viral vector [21]. The particularity of the 12S E1A gene is its ability to stimulate rodent cell growth without tumorigenesis [22]. However, the R28 progenitor cell line was found to predominantly express retinal ganglion cell and glial cell markers [23]. In recent years, R28 retinal precursor cells have been used for numerous studies, including in vitro toxicity [24,25], neuroprotection [26,27] and retinal transplantation [28].

The aim of this study was to elucidate the pathological mechanisms underlying neuroretinal R28 cell death after exposure to glutamate and hydrogen peroxide (H_2_O_2_) in order to develop new therapeutic approaches for oxidative stress-induced retinal diseases, including glaucoma.

## 2. Materials and Methods

### 2.1. R28 Cell Culture

R28 cells were cultured in T75 flasks (Thermo Scientific, Roskilde, Denmark) containing DMEM/Ham’s F-12 liquid medium with stable glutamine (Bio&SELL GmbH, Nuremberg, Germany) supplemented with 10% fetal bovine serum (FBS) (Gibco, Paisley, UK) and 1% penicillin–streptomycin (Gibco, Schwerte, Germany). Cells were passaged every 2 days and incubated at 37 °C in 5% CO_2_ to reach confluence.

### 2.2. Glutamate- and Hydrogen Peroxyde (H_2_O_2_)-Induced Toxicity in R28 Cells

**Figure 1:**
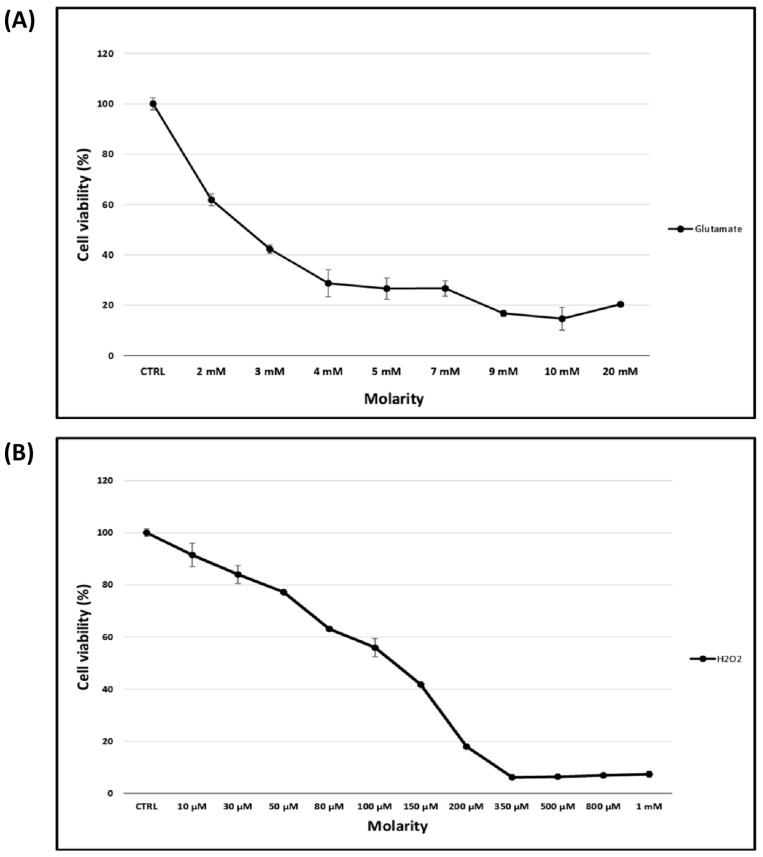
Evaluation of the glutamate- and H_2_O_2_-induced toxicity in R28 cells in vitro. R28 cells were cultured in medium and exposed to concentrations of glutamate ranging from 0 to 20 mM (**A**) and H_2_O_2_ ranging from 0 to 1 mM (**B**) for 24 h at 37 °C. The cell viability was determined by the MTS assay. The data showed that glutamate and H_2_O_2_ could induce a dose-dependent decrease in R28 cell viability in vitro. A cell viability of 42% and 56% was observed after exposure to 3 mM of glutamate and 100 µM of H_2_O_2_, respectively.

The R28 cells (4 × 10^4^ cells) were placed in 96-well plates and incubated with the DMEM/Ham’s F-12 liquid medium supplemented with 10% FBS and 1% penicillin–streptomycin for 24 h at 37 °C. Subsequently, the culture medium was replaced with a red phenol-free DMEM/F-12 medium (Gibco, Paisley, UK) supplemented with 10% FBS with different concentrations of glutamate (0, 2, 3, 4, 5, 7, 9, 10 and 20 mM) and H_2_O_2_ (0, 10, 30, 50, 80, 100, 150, 200, 350, 500, 800 and 1000 µM) and without any additive as a control. Thereafter, the R28 cells were incubated for 24 h at 37 °C (*n* = 4). The R28 cells’ viability was determined using the MTS assay. The MTS reagent (CellTiter 96^®^ AQueous One Solution Cell Proliferation Assay; Promega Corporation, Madison, WI, USA) consists of tetrazolium compounds and an electron coupling reagent (phenazine ethosulfate). The experiment is based on the ability of viable cells to reduce the reagent to a soluble compound called formazan. The amount of produced formazan is therefore measured by absorbance at 490 nm and is directly proportional to the number of living cells in the culture. Following treatment with glutamate and H_2_O_2_, 20 µL of MTS reagent was added to each well and incubated for 2 h at 37 °C in 5% CO_2_. Afterwards, the absorbance was measured on a Multiscan Ascent photometer (Thermo Fisher Scientific, Rockford, IL, USA). A cell viability of about 42% (*p* = 2.1 × 10^−4^) was observed after exposure to 3 mM of glutamate and about 56% (*p* = 1.8 × 10^−3^) after exposure to 100 µM of H_2_O_2_, and this was considered a stress parameter for further analysis (see Figure 1).

### 2.3. Mass Spectrometry Analysis

#### 2.3.1. Sample Preparation and In-Solution Digestion

For the mass spectrometry (MS) analysis, 5 × 10^6^ cells were placed in 6-well plates and exposed to either 3 mM of glutamate or 100 µM of H_2_O_2_ or left untreated as a control (*n* = 3 per group). After 24 h, the cells were morphologically examined by light microscopy (Leica Microsystems GmbH, Wetzlar, Germany), harvested by scraping and stored at −20 °C in 1.5 mL tubes. Prior to homogenization, the tubes were filled with 300 µL of Tissue Protein Extraction Reagent, T-PER (Thermo Fisher Scientific, Rockford, IL, USA), as well as a 1% protease and phosphatase inhibitor cocktail (Thermo Fisher Scientific, Rockford, IL, USA), and subsequently subjected to homogenization with a Bullet Blender^®^ Storm 24 homogenizer (Next Advance, Inc., New York, NY, USA) as described in our previous studies [29,30]. The samples were then centrifuged, and the supernatant containing the proteins was collected in new 1.5 mL tubes. Proteins were extracted from supernatants containing T-PER buffer and introduced into 200 µL of phosphate-buffered saline (PBS) using an Amicon 3 kDa centrifugal filter device (Millipore, Billerica, MA, USA), and protein measurement was performed using the Pierce BCA protein assay kit (Thermo Fisher Scientific, Rockford, IL, USA) according to the manufacturer’s protocol. After that, 10 µg of protein from each sample was collected and dried using a vacuum concentrator (Eppendorf AG, Hamburg, Germany) and then stored at −20 °C for further analysis.

The tryptic digestion of proteins was performed as described in our previous studies [31,32]. In brief, protein fractions were dissolved in 20 μL of 10 mM ammonium bicarbonate (ABC) and sonicated for 10 min on ice. Subsequently, 6 μL of 100 mM dithiothreitol (DTT) in 10 mM ABC was added and incubated for 30 min at 56 °C. After that, 6 μL of 200 mM iodoacetamide (IAA) in 10 mM ABC was added and incubated for 30 min at RT in the dark. The reduced and alkylated proteins were digested overnight with trypsin solution (Promega, Madison, WI, USA) at a sample ratio of 1:20 at 37 °C. The next day, the digestion was quenched with 10 μL of 0.1% formic acid (FA), evaporated in a vacuum concentrator at 30 °C to dryness and stored at −20 °C. Prior to the LC-MS/MS analysis, tryptic peptides were purified with SOLAμ™ HRP SPE spin plates (Thermo Fisher Scientific, Rockford, IL, USA) following the manufacturer’s protocol.

#### 2.3.2. LC-MS Analysis

The liquid chromatography mass spectrometry (LC-MS) analysis was performed using a Hybrid Linear Ion Trap–Orbitrap MS system (LTQ–Orbitrap XL; Thermo Fisher Scientific, Rockford, IL, USA) coupled to an EASY-nLC 1200 system (Thermo Fisher Scientific, Rockford, IL, USA) [29,30,33]. The peptide separation was performed using an analytical column (75 µm × 50 cm, nanoViper, C18, 2 µm, 100 Å) (Thermo Fisher Scientific, Rockford, IL, USA) using a 60 min gradient for the elution of peptides as follows: 15–40% B (0–30 min), 40–60% B (30–35 min), 60–90% B (35–45 min) and 90–10% B (45–60 min). Solvent A was 0.1% formic acid (FA) in water and solvent B was 0.1% FA in 80% acetonitrile (ACN). The LTQ–Orbitrap was operated in positive ionization and in the data-dependent acquisition mode to automatically switch from Orbitrap-MS to LTQ-MS/MS acquisition. The recording of full-scan MS spectra (from *m*/*z* 300 to 2000) was completed in the orbitrap with a resolution of 30,000 at 400 *m*/*z* and a target gain control setting of 1 × 10^6^ ions. The polydimethylcyclosiloxane ions *m*/*z* 445.120025 were used as the lock mass for the internal calibration. The dynamic exclusion mode was adjusted as follows: number of repetitions = 2; repetition time = 30 s; exclusion list size = 100; exclusion time = 90 s; and exclusion mass width = ±20 ppm. The five most intense precursor ions were selected for additional fragmentation in the ion trap with collision-induced decay (CID) fragmentation using the normalized collision energy set at 35%.

#### 2.3.3. Protein Identification and Quantification

The acquired LC-MS dataset was analyzed with MaxQuant v. 2.1.3.0 (Max Planck Institute for Biochemistry, Martinsried, Germany). The tandem MS spectra were searched against Uniprot databases for *Homo sapiens*, *Rattus norvegicus* and *Mus musculus* using standard parameters: a peptide mass tolerance of ±30 ppm, a fragment ion tolerance of 0.5 Da, tryptic cleavage, maximum 2 missing cleavage motifs, carbamidomethylation as a fixed modification, and acetylation (N-terminal protein) and oxidation as variable modifications. The protein annotation research was conducted with three different databases to allow maximal identification of the peptides because of the limited annotation of the *Rattus norvegicus* database (8172 proteins) compared to the *Mus musculus* (17,137 proteins) and *Homo sapiens* (207,304 proteins) databases. In addition, the use of several databases was reported to allow higher and more complete peptide identification [29,34,35]. Moreover, it has been shown that rat and mouse organisms share a gene homology estimated at 83–100%, and this is approximately 66–82% between rat and human organisms [36]. The peptide and protein identification was performed using a target-decoy-based false discovery rate (FDR) < 1%.

#### 2.3.4. Statistical Analysis and Bioinformatics

The data generated by MaxQuant (“proteinGroups.txt”) were used for statistical analysis with Perseus software version 1.6.13.0 (Max Planck Institute of Biochemistry, Martinsried, Germany). The LFQ intensities of the identified proteins were log_2_-transformed and filtered for possible contaminants and misassigned protein identifications. The identified proteins needed to be recovered in the three biological replicates in at least one study group to be considered for further analysis. Missing protein intensity values were imputed based on the normal distribution of the data (width: 0.3, downshift: 1.8). The significantly changed protein expressions between the control and treated groups (with glutamate and H_2_O_2_) were identified by a Student’s two-sided *t*-test with *p* values < 0.05. To illustrate the heat map of differentially expressed proteins between the different groups, a hierarchical clustering based on the Euclidean distance was performed. In addition, the functional annotation and the signalling pathway analyses were carried out using Ingenuity Pathway Analysis software v. 1-04 (IPA, Ingenuity QIAGEN; Redwood City, CA, USA) [29,34].

### 2.4. Microarray Analysis

#### 2.4.1. Slide and Sample Preparation

Microarray manufacturing was performed in our lab using a noncontact microarray printer (SciFLEXARRAYER S3; Scienion, Berlin, Germany). The HTRA2 antibodies (Proteintech GmbH; Manchester, UK) were spotted onto nitrocellulose-coated microarray slides (AVID Oncyte, NC 16 Pad slides; Grace Bio-Labs, Bend, OR, USA) in triplicate. An amount of 40 µg of cell lysates from R28 cells exposed to glutamate and H_2_O_2_ as well as untreated controls were prepared in labeling buffer (0.05 M sodium borate buffer, pH 8.5) and subsequently labeled with a fluorescent dye (DyLight 650 NHS Ester; Thermo Fisher Scientific, Rockford, IL, USA). Samples were incubated with 1 µL of dye overnight at 4 °C in the dark. In addition, a negative control containing only the labeled buffer as well as a positive control comprising a pooled sample were also included in this experiment. To stop the labeling reaction, 10 µL of quenching solution (Tris-HCl, pH 8.8) was added to the samples and incubated for 30 min at room temperature in the dark. Unbound dye was removed using Zeba desalting plates (Zeba Spin Desalting Plates, 7k MWCO; Thermo Fisher Scientific, Rockford, IL, USA) according to the manufacturer’s protocol.

#### 2.4.2. Microarray Incubation and Image Acquisition

The prepared slide was mounted in a 16-well incubation chamber (ProPlate Multiwell Chambers; Grace Bio-Labs, Bend, OR, USA) and subsequently incubated with blocking buffer (Super G; Grace Bio-Labs, Bend, OR, USA) for 1 h at 4 °C to improve the signal-to-noise ratio. Then, the blocking buffer was discarded, and the slide was washed three times with PBST (phosphate-buffered saline; PBS with 0.5% Tween-20). Afterwards, the slide was incubated with 40 µg of subgroup-labeled sample proteins (*n* = 3 per subgroup) overnight at 4 °C in a cooling shaker. In addition to the labeled buffer, another group was incubated with only PBS as an additional negative control. After incubation, the residual sample was removed, and the slide was washed twice with PBST and twice with ultrapure water. Finally, the slide was dried for 2 min in a SpeedVac at 30 °C. Immediately after drying, the slide was imaged using a CCD camera-based array reader (SensoSpot; Sensovation, Radolfzell, Germany). Then, the slide was scanned at 25 ms and 100 ms exposure times in the red channel. The images were saved as 16-bit TIF files.

#### 2.4.3. Data Processing and Statistical Analysis

The quantification of spot intensity was performed using Imagene software (Imagene 5.5; BioDiscovery Inc., Los Angeles, CA, USA). Spots that did not meet quality control criteria were flagged and removed from further analysis. Prior to statistical evaluation, the microarray data were preprocessed. Thus, the local background intensity was removed from the spot intensity to calculate the net signal intensities, and the triple-spot signals were averaged. Afterwards, a graphical presentation was produced and a two-sided *t*-test with a *p* value < 0.05 was performed using statistica version 13 (Statsoft; Tulsa, OK, USA) to identify significant changes in protein expression between the control and stressed groups (glutamate and H_2_O_2_).

## 3. Results

### 3.1. Glutamate- and H_2_O_2_-Induced Toxicity in R28 Cells

**Figure 2:**
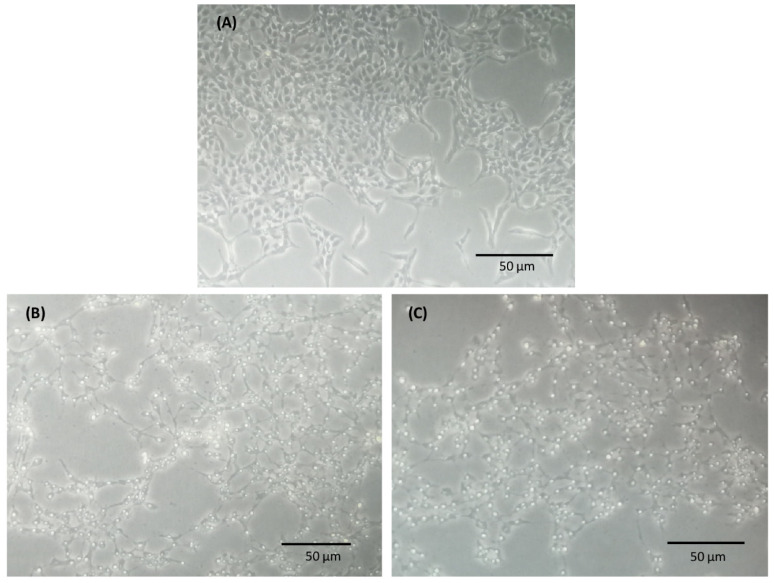
Morphological changes in R28 cells due to glutamate- and H_2_O_2_-induced toxicity. The R28 cells were cultured in a medium without stressor (control) (**A**) or exposed to 3 mM of glutamate (**B**) and 100 µM of H_2_O_2_ (**C**) for 24 h at 37 °C. After exposure to 3 mM of glutamate and 100 µM of H_2_O_2_, marked morphological alterations and the degeneration of R28 cells were observed.

To assess glutamate- and H_2_O_2_-induced toxicity, R28 cells were exposed to different concentrations of glutamate and H_2_O_2_ for 24 h at 37 °C. The viability of the R28 cells was determined using the MTS assay according to the manufacturer’s protocol. A decrease in cell viability in a concentration-dependent manner of the stress factors (glutamate and H_2_O_2_) was observed (see Figure 1). After 24 h, a cell viability of 42% was observed after exposure to 3 mM of glutamate, and 56% was observed after exposure to 100 µM of H_2_O_2._ These parameters were considered stress conditions for further experiments. Prior to cell homogenization and MS analysis, a microscopic analysis was performed under a light microscope (Leica Microsystems GmbH, Wetzlar, Germany). This showed the morphological alteration and degeneration of the R28 cells after 24 h of exposure to 3 mM of glutamate and 100 µM of H_2_O_2_ (see Figure 2).

### 3.2. LC-MS Analysis

To explore the molecular mechanisms induced by glutamate and H_2_O_2_ on the R28 cells, we performed a quantitative LC-ESI-MS analysis of the cells after exposure to 3 mM of glutamate and 100 µM of H_2_O_2_, as well as without any treatment, considered the control, for 24 h at 37 °C (complete data in Supplementary File S1). A total of 2249 and 2252 proteins were identified in the R28 cells after exposure to glutamate and H_2_O_2_, respectively, compared to the control (FDR < 1%). The majority of the proteins in both groups (glutamate and H_2_O_2_) were identified in the *Rattus norvegicus* database (1002 proteins), followed by the *Mus musculus* (707 and 706 proteins) and *Homo sapiens* (538 and 542 proteins) databases (see Supplementary Files S2 and S3). The statistical analysis of proteomic data revealed that 193 and 311 proteins were significantly altered in the cells exposed to glutamate and H_2_O_2_, respectively, compared to the control cells (FDR < 1%; *p* < 0.05).

#### 3.2.1. Proteomic Alterations in R28 Cells Exposed to Glutamate

**Figure 3:**
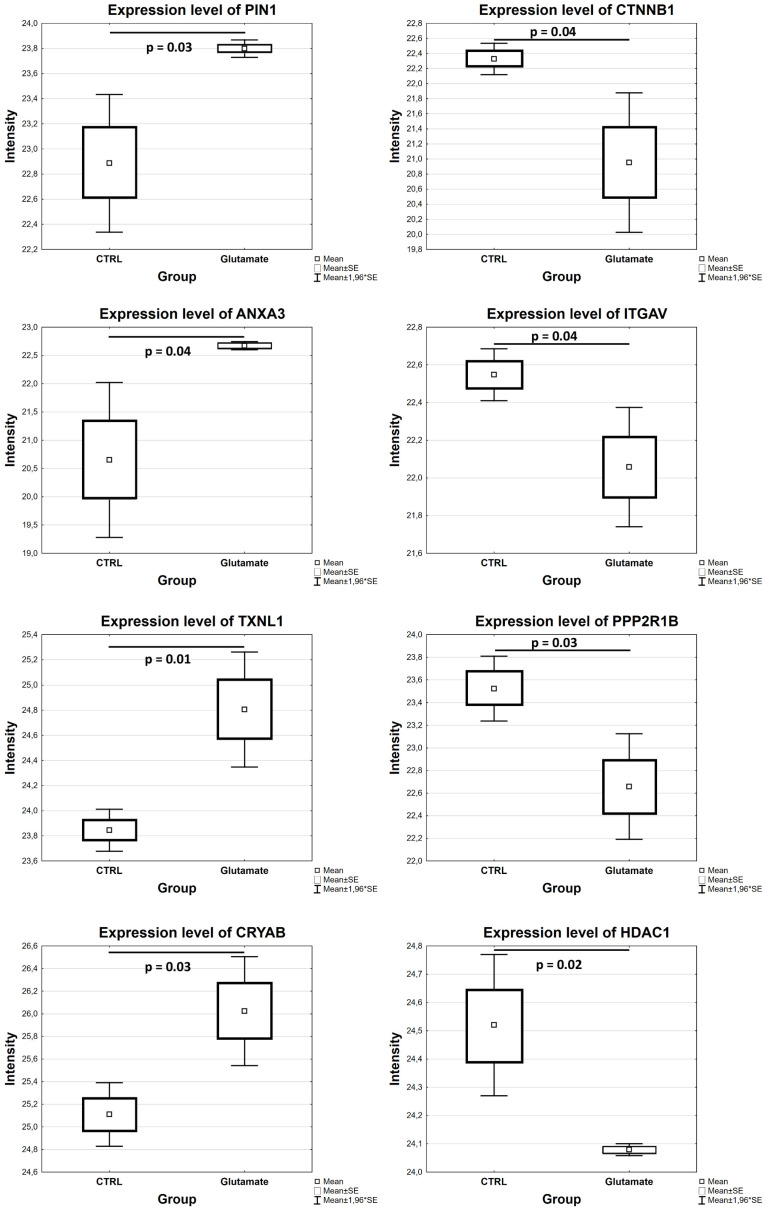
Differentially abundant proteins in R28 cells exposed to glutamate compared to control (*p* < 0.05). Box plots show expression levels of selected proteins from MS data analysis.

Glutamate-induced toxicity on R28 cells led to the differential expression of 193 proteins. Thus, 80 proteins were highly expressed, while 113 proteins were found in low abundance in the cells exposed to glutamate compared to the untreated cells (*p* < 0.05; see Supplementary File S2). In particular, proteins like peptidyl-prolyl cis–trans isomerase NIMA-interacting 1 (PIN1), annexin A3 (ANXA3), alpha-crystallin B chain (CRYAB), thioredoxin-like protein 1 (TXNL1) and heat shock protein 70 (Hspa9/Hsp70) were upregulated, while catenin beta-1 (CTNNB1), integrin alpha-V (ITGAV), serine/threonine-protein kinase mTOR (MTOR), serine/threonine-protein phosphatase 2A 65 kDa regulatory subunit A beta isoform (PPP2R1B) and histone deacetylase 1 (HDAC1) were hardly expressed in the cells exposed to glutamate (*p* < 0.05; see Figure 3 and Supplementary File S2).

#### 3.2.2. Proteomic Alterations in R28 Cells Exposed to H_2_O_2_

**Figure 4:**
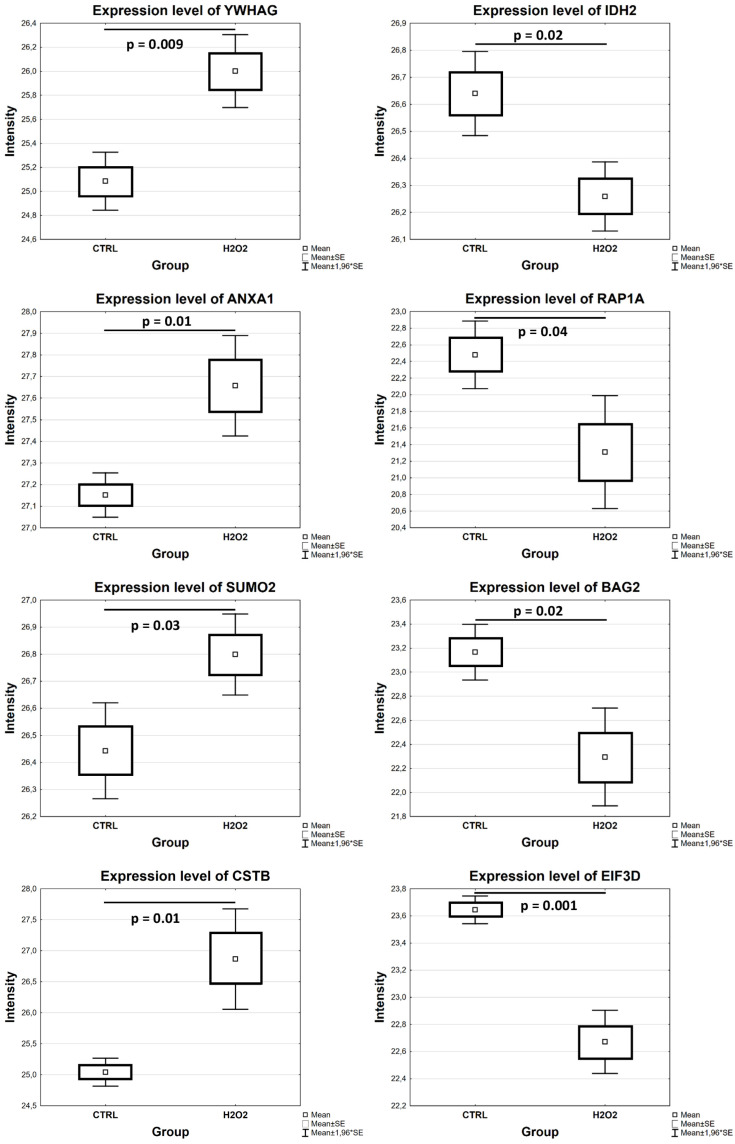
Differential expression of significantly altered proteins in R28 cells after exposure to H_2_O_2_ in comparison to control cells (*p* < 0.05). Box plots illustrate the expression levels of some proteins resulting from the analysis of MS data.

**Figure 5:**
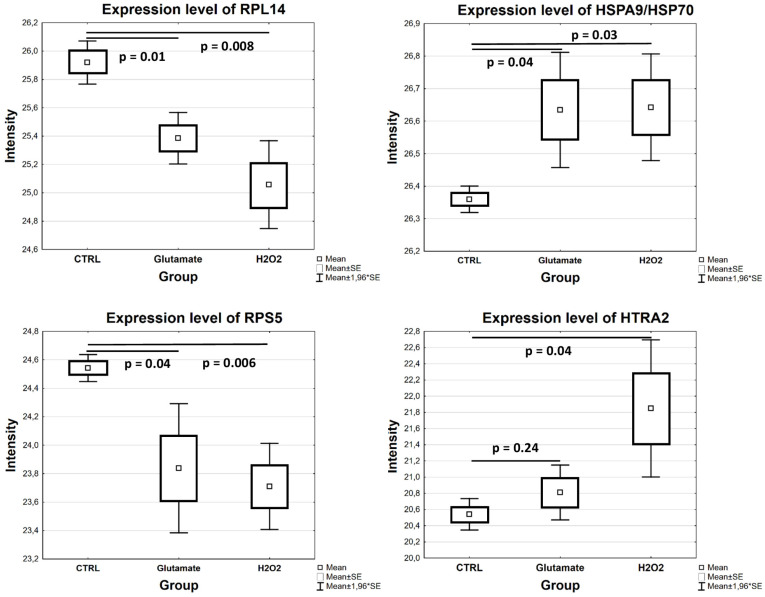
Differential expression levels of certain proteins in MS dataset. The box plot highlights the similar expression levels of altered proteins in R28 cells exposed to glutamate and H_2_O_2_ compared to CTRL (*p* < 0.05). Similar to the H_2_O_2_ group, HTRA2 was found to be highly expressed in the glutamate group compared to the CTRL, but this was not statistically significant (*p* > 0.05).

A total of 311 proteins were significantly altered in the cells exposed to H_2_O_2_ (*p* < 0.05; see Supplementary Files S3). In particular, 176 proteins, including isocitrate dehydrogenase (IDH2), 60S ribosomal proteins (RPLs), 40S ribosomal proteins (RPSs), BAG family molecular chaperone regulator 2 (BAG2), Ras-related protein Rap-1A (RAP1A), the signal transducer and activator of transcription 1 and 3 (STAT1 and STAT3), and eukaryotic translation initiation factor 3 subunit D (EIF3D), were downregulated, whereas 135 proteins, including high-temperature-requirement protein A2 (HTRA2), 14-3-3 protein gamma (YWHAG), thioredoxin-like protein 1 (TXNL1), cystatin-B (CSTB), heat shock protein 70 (Hspa9/Hsp70), annexin A1 (ANXA1) and small ubiquitin-related modifiers 2 and 4 (SUMO2 and SUMO4), were upregulated in the cells treated with H_2_O_2_ compared to the control (*p* < 0.05; see Figure 4 and Figure 5 and Supplementary File S3).

#### 3.2.3. Top Canonical Pathways as Well as Diseases and Biological Functions Associated with the Significantly Differentially Expressed Proteins in R28 Cells after Exposure to Glutamate and H_2_O_2_

**Figure 6:**
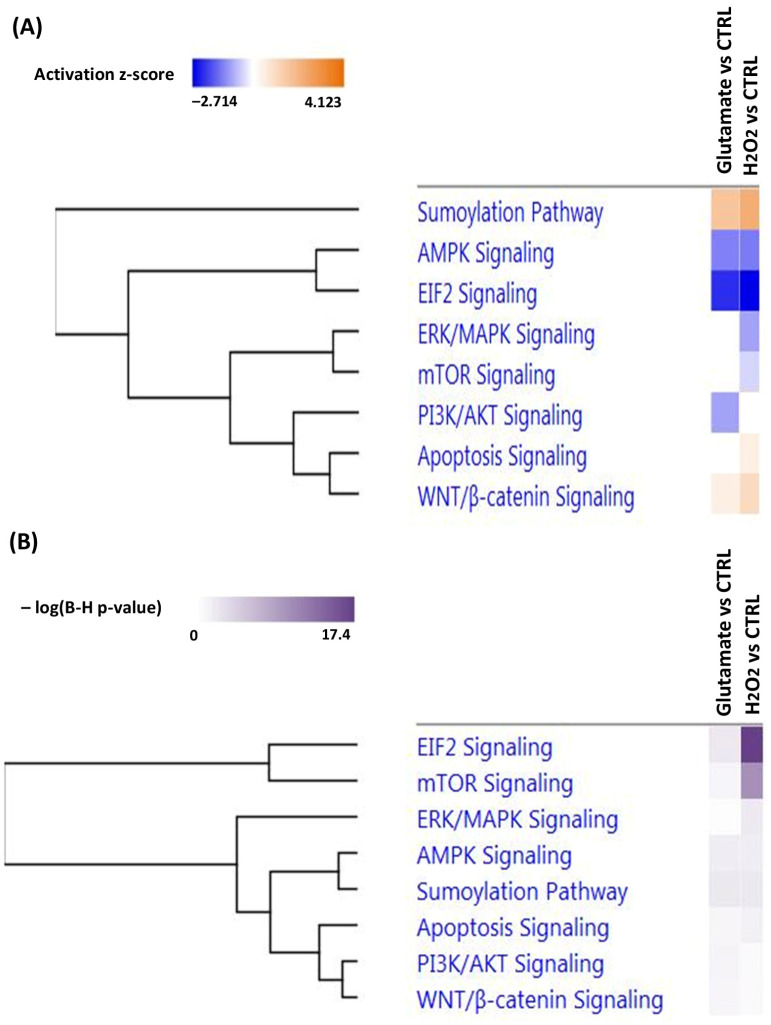
Top enriched canonical pathways. The hierarchical clustering shows the most affected canonical pathways related to significantly altered proteins in the R28 cells exposed to glutamate and H_2_O_2_ compared to the control. (**A**) exhibits the regulation of canonical pathways and biological functions. The negative regulation is depicted by the blue color and the positive regulation by the orange color based on activation z-scores of <−2 and >4. (**B**) was determined by the default IPA threshold [−log (*p*-value) > 0] between significantly changed proteins identified in our datasets and molecules in the respective pathways.

**Figure 7:**
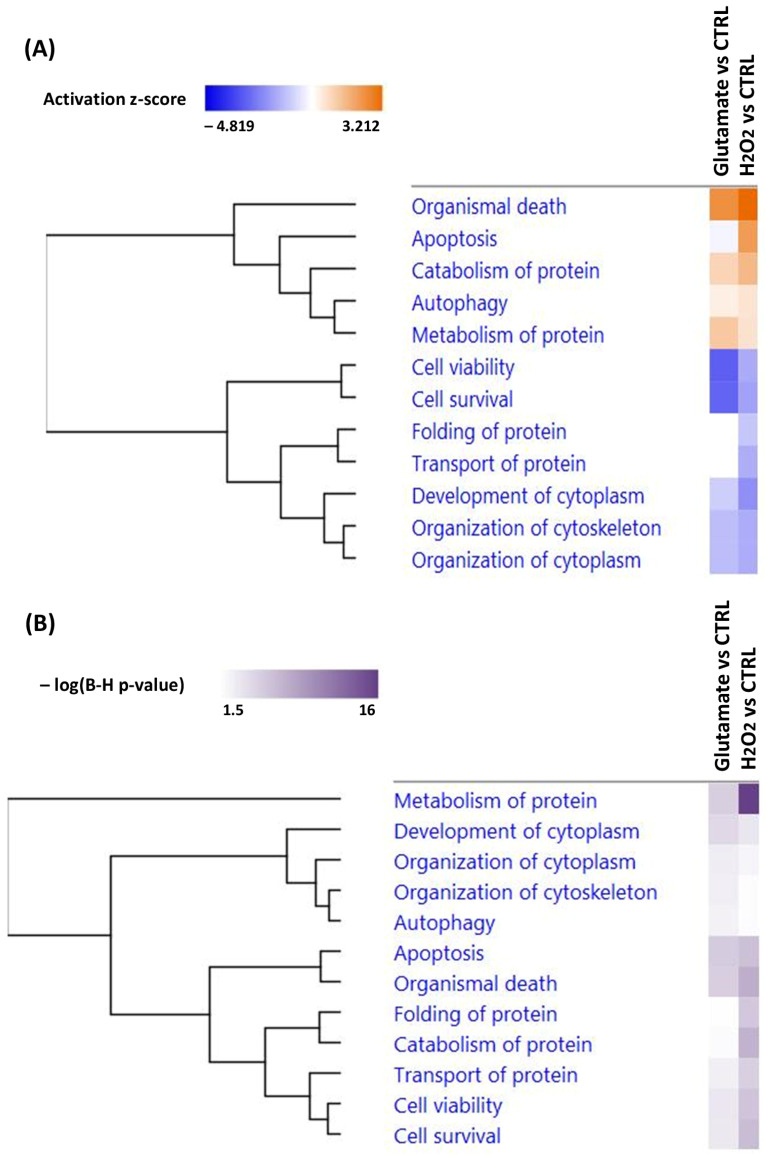
Top significantly affected biological functions in R28 cells after exposure to glutamate and H_2_O_2_ compared to untreated cells. (**A**) shows the regulation of biological functions. The downregulation is highlighted in blue and the upregulation in orange based on activation z-scores of <−4 and >3. (**B**) represents the hierarchical clustering of biological functions according to the −log10 (*p*-value) difference between significantly altered proteins identified in our datasets and molecules in the respective pathways.

To investigate the biological processes involved in the decrease in R28 cell viability after exposure to glutamate and H_2_O_2_, we performed an ingenuity pathway analysis (IPA) to identify the most affected canonical pathways (see Figure 6) as well as diseases and biological functions (see Figure 7) related to differentially expressed proteins in both study groups. Interestingly, glutamate treatment inhibited phosphatidylinositol 3-kinases/protein kinase B (PI3K/AKT) signaling (z-score = −1). In contrast, beside apoptosis signaling (z-score = 0.447), which was activated, the mammalian target of rapamycin (mTOR) signaling (z-score = −0.447) and extracellular signal-regulated kinases/mitogen-activated protein kinase (ERK/MAPK) signaling (z-score = −1) pathways were inhibited by H_2_O_2_-induced oxidative stress in the R28 cells. However, exposure to glutamate and H_2_O_2_ showed similarities in the inhibition of the expression of eukaryotic initiation factor 2 (EIF2) signaling (z-score = −2.236 and −2.713) and AMP-activated protein kinase (AMPK) signaling (z-score = −1.341 and −1.414), as well as the activation of the sumoylation signaling pathway (z-score = 1.633 and 2.236) and WNT/β-catenin signaling (z-score = 0.447 and 1 for glutamate and H_2_O_2_, respectively).

Strikingly, the biological functions associated with differentially expressed proteins in the cells exposed to H_2_O_2_ included the activation of networks involved in apoptosis (z-score = 2.14) and the inhibition of clusters involved in the folding of proteins (z-score = −1.09) and transport of proteins (z-score = −1.54). However, an increase in organismal death (z-score = 2.41 and 3.21 for glutamate and H_2_O_2_, respectively) and the catabolism of proteins (z-score = 0.92 and 1.51) as well as a decrease in cell survival (z-score = −2.93 and −1.79) and cell viability (z-score = −3.06 and −1.78) were observed in the R28 cells exposed to both stressors (see Figure 7A).

### 3.3. Microarray Analysis

**Figure 8:**
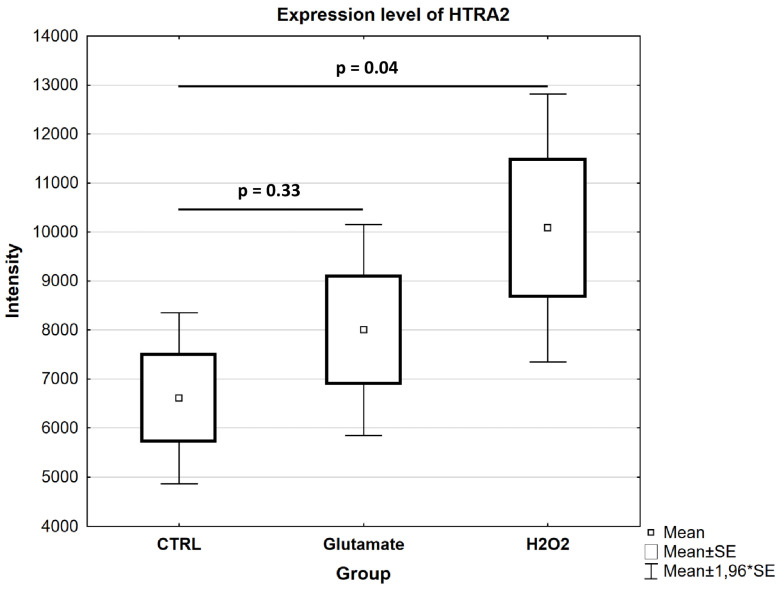
Validation of the marker protein HTRA2 using microarrays. The box plot depicts the expression level of the HTRA2 protein, validated with microarrays. Showing similar trends to the results obtained by MS analysis, HTRA2 was identified with significantly higher expression in the cells exposed to H_2_O_2_ compared to the untreated CTRL (*p* < 0.05). The HTRA2 protein was also identified in high abundance in the glutamate-exposed group compared to the control, but this was not statistically significant (*p* > 0.05).

For the validation of the significantly altered proteins identified by mass spectrometry, we performed a quantitative analysis of the marker protein HTRA2 using microarrays in the different study groups (CTRL, glutamate and H_2_O_2_). The statistical analysis of the data revealed that HTRA2 was found in significantly high abundance in the H_2_O_2_-exposed cells compared to the control (*p* = 0.04; see Figure 8), and this was in agreement with the results obtained by mass spectrometry (see Supplementary File S3). Similar to the MS results, the data analysis also showed an increase in HTRA2 expression in the cells exposed to glutamate (see Figure 8), but this was not significant (*p* = 0.33; see Figure 5 and Supplementary File S2).

## 4. Discussion

In recent years, the establishment of disease models such as glaucoma models has emerged as an effective tool for the understanding of pathological mechanisms and for the investigation of innovative therapeutic approaches to prevent RGC loss. Thus, the aim of this study was to propose a cell culture model based on neuroretinal R28 cells for the development of new therapeutic approaches for retinal diseases, such as glaucoma. To this end, we investigated the ability of glutamate and hydrogen peroxide (H_2_O_2_) to induce neuroretinal cell death in vitro. However, we were able to show a decrease in R28 cell viability in a concentration-dependent manner for both stressors (see Figure 1).

Glutamate-induced toxicity in R28 cells was characterized by the differential expression of 193 proteins, of which 80 were highly abundant and 113 were downregulated (see Supplementary File S2). These modifications were also accompanied by profound perturbations in signaling pathways (see Figure 6A) and biological functions (see Figure 7A) crucial for cellular homeostasis. Accordingly, our data revealed an inhibition of PI3K/AKT signaling (z-score = −1), which was coupled with a decrease in proteins like catenin beta-1 (CTNNB1), integrin alpha-V (ITGAV), serine/threonine-protein kinase mTOR (MTOR), serine/threonine-protein phosphatase 2A 65 kDa regulatory subunit A beta isoform (PPP2R1B) and Ras-related protein Rap-1A (RAP1A) in the R28 cells exposed to glutamate compared to the control cells (see Supplementary Figure S1A). This finding was in agreement with previous studies from other groups in which the inhibition of PI3K/AKT signaling was observed in retinal tissues in animal models of glaucoma [37,38]. However, phosphatidylinositol 3-kinase (PI3K) is an enzyme that catalyzes the phosphorylation of the inositol ring of membrane-localized phosphoinositides, thus generating phosphorylated phosphoinositides like phosphatidylinositol-3,4,5-triphosphate (PI(3,4,5)P3) [39]. These phosphorylated phosphoinositides can induce the phosphorylation of the AKT protein at the activation loop (Thr308) [40]. However, the complete activation of the AKT protein requires phosphorylation at two sites, namely the activation loop (Thr 308) and the hydrophobic motif (Ser 473), which is phosphorylated by the rictor–mTOR complex [41]. Then, fully activated AKT can phosphorylate many substrates in the cytoplasm and nucleus, which plays an important role in cellular processes such as apoptosis [42], the cell cycle [43], cell proliferation [44], cell energy metabolism [45] and gene regulation [46]. Interestingly, RAP1A, which is a member of the RAS protein family, and ITGAV were also reported to be involved in the regulation of PI3K/AKT signaling [47,48,49,50]. However, it has been shown that inhibition of PI3K/AKT signaling can lead to cell death by apoptosis [51]. In contrast, the activation of PI3K/AKT signaling is able to suppress apoptosis and promote the increased viability of RGCs in glaucoma [37,38,52].

The proteomic data obtained from the H_2_O_2_-exposed R28 cells revealed an activation of apoptosis signaling (see Figure 6A), which is a well-described pathological event in glaucoma [53,54]. However, the activation of apoptosis signaling was particularly characterized by an increase in HTRA2 protein expression (see Supplementary Figure S1C). Thus, the significant increase in HTRA2 expression was validated by microarray technology in the cells exposed to H_2_O_2_ (see Figure 8). In particular, HTRA2 is a multifunctional protein that plays an important role in the induction of apoptosis in cells under stress [55,56]. Moreover, it has been shown that the overexpression of HTRA2 induces cell death in the injured retina [57,58]. In addition, we were able to show in our previous studies that the inhibition of HTRA2 protease activity by small synthetic CDR1 peptides can increase RGCs’ viability in vitro [31,59]. Based on these results, we could suggest a possible involvement of HTRA2 in the apoptotic process related to glaucoma. Another protein related to apoptosis signaling was CDK1, which was found to be downregulated in the R28 cells exposed to H_2_O_2_. CDK1 is a protein involved in the regulation of cellular processes such as the cell cycle and transcription [60,61]. However, some studies have shown that CDK1 inhibition in cells under stress might lead to cell cycle dysfunction and apoptosis [62,63].

The H_2_O_2_-induced oxidative stress in the R28 cells was further demonstrated by the inhibition of mTOR signaling (see Figure 6A), associated with decreased expression of ribosomal proteins such as 40S ribosomal proteins S (RPS13, RPS14, RPS27L, etc.) and proteins responsible for translation initiation like EIF3A and EIF3D (see Supplementary Figure S1D). Interestingly, mTOR is a serine/threonine kinase that plays a crucial role in several cellular processes, including cell growth and proliferation, cytoskeleton organization, transcription, protein synthesis and ribosomal biogenesis [64]. In addition, various reports have suggested that mTOR can positively regulate different steps of ribosome biogenesis, such as ribosomal RNA transcription and processing, as well as ribosomal protein synthesis [65,66]. Nevertheless, it has been shown that increased oxidative stress is able to suppress the mTOR signaling pathway, resulting in impaired ribosomal functions, which could lead to apoptosis [67,68]. In addition, the impairment of ribosomal biogenesis could lead to endoplasmic reticulum (ER) stress [69], which is one of the pathological events resulting in cell death in glaucoma [70] as well as in diabetic retinopathy (DR) and age-related macular degeneration (AMD) [71]. These arguments could be supported by the fact that an inhibition of biological functions involved in the folding of proteins and the transport of proteins, as well as an activation of the catabolism of proteins, was shown in the IPA analysis (see Figure 7A).

In addition to mTOR, the proteomic data also showed an inhibition of extracellular regulated kinase (ERK)/mitogen-activated protein kinase (MAPK) signaling (see Figure 6A) in the cells exposed to H_2_O_2_. Our data revealed that, except for the YWHAG and CRK proteins, which were upregulated, the majority of the proteins clustered in this pathway were downregulated (see Supplementary Figure S1E). These included serine/threonine-protein phosphatase 2A proteins (PPP2R1B and PPP2R2A), Ras-related protein Rap-1A (RAP1A) and Ras-related C3 botulinum toxin substrate 1 (RAC1), as well as signal transducer and activator of transcription 1 and 3 (STAT1 and STAT3). However, the ERK/MAPK signaling pathway is a pathway consisting of signaling cascades that play an important role in the control of cellular processes such as cell proliferation, cell survival and apoptosis [72]. In addition, H_2_O_2_-induced oxidative stress has been reported to inhibit serine/threonine-protein phosphatase 2A protein activity, which modulates the Raf-1 kinase-induced activation of ERK/MAPK signaling [73,74,75]. Moreover, the role of ERK/MAPK is still controversial, as its activation can exert either pro-apoptotic or anti-apoptotic functions in mammalian cells [76]. Nevertheless, many studies have shown the involvement of ERK/MAPK signaling in the pathological process of glaucoma [77,78], but its exact role remains to be elucidated.

Besides the differentially expressed signaling pathways in the cells exposed to either glutamate or H_2_O_2_, the data analysis showed some similarities in the expressions of certain metabolic pathways in the cells exposed to both stressors. Thus, exposure to glutamate and H_2_O_2_ showed similarities in the inhibition of the EIF2 signaling pathway (see Figure 6A). EIF2 is a translational factor that induces and regulates the translation of mRNA into proteins [79]. However, the phosphorylation of EIF2 in response to oxidative stress has been reported to reduce its ability to induce translation, resulting in a decrease in protein synthesis [80], which could lead to apoptosis [81,82]. Accordingly, our data showed a decrease in the expression of proteins associated with this signaling pathway, like 60S ribosomal proteins L (RPL14, RPL21, etc.) and 40S ribosomal proteins S (RPS5, RPS24, etc.) (see Supplementary Figure S1F), which are found to be key players in protein synthesis. In addition, an increased level of phosphorylated EIF2 is associated with ER stress-induced RGC death in glaucoma [83]. Moreover, Liu Yang et al. (2016) showed that blocking EIF2 phosphorylation might promote the neuroprotection of RGCs and preserve visual function in glaucoma [84].

Another similarity in both groups was shown by the inhibition of AMPK signaling (see Figure 6A) and the decrease in its associated proteins, including PFKM, PFKP, PPP2R1B, PPP2R2A, RAB6A, RAB8A and PRKAR2A (see Supplementary Figure S1G). However, AMPK is an enzyme that plays an essential role in biological processes such as glucose and lipid metabolism, transcription, cell growth and cell polarity [85]. In line with our results, Duygu Sag et al. (2008) showed that pro-inflammatory stimuli are able to inhibit AMPK activity in macrophages [86]. In addition, the inhibition of AMPK signaling could promote mitochondrial metabolism dysfunction, resulting in decreased ATP production [87]. Interestingly, the majority of proteins (PFKM, PFKP, PPP2R1B, PPP2R2A and PRKAR2A) connected to AMPK signaling were found to be key players in glycolysis, which represents the first step in ATP biosynthesis [88]. Thus, the downregulation of these proteins may suggest a decrease in energy metabolism in the R28 cells exposed to glutamate and H_2_O_2_. Furthermore, a decline in energy metabolism has been shown to be involved in neurodegenerative diseases such as Alzheimer’s and Parkinson’s diseases [89], as well as retinal diseases, such as DR [90], AMD [91] and glaucoma [92].

In the same way, the data also showed an activation of the sumoylation pathway in the R28 cells exposed to both stressors (see Figure 6A). Sumoylation is a post-translational modification characterized by the conjugation of small ubiquitin-like modifier (SUMO) proteins to the lysine residues of target proteins, thereby regulating several biological functions such as the cell cycle, transcription, subcellular transport and DNA repair [93]. Similar to ubiquitination, sumoylation is involved in the targeting and degradation of proteins by the ubiquitin–proteasome system (UPS) [94]. Moreover, it might act as a sensor regulating homeostasis in the ER and mitochondria in cells under stress [95,96]. Nevertheless, Feliogioni et al. (2011) showed that the over-activation of the sumoylation pathway can induce cell death by apoptosis [97]. In addition, the sumoylation pathway has been associated with the pathological mechanisms of neurodegenerative diseases like Alzheimer’s, Parkinson’s and Huntington’s diseases [98,99,100]. Furthermore, an increased level of sumoylated proteins was shown in an ex vivo model of retinal degeneration [101], which could suggest a possible involvement of the sumoylation pathway in the neurodegenerative events observed in glaucoma.

Another striking feature in the R28 cells stressed by glutamate and H_2_O_2_ was the activation of WNT/β-catenin signaling (see Figure 6A), which was mediated by the downregulation of its related proteins, namely casein kinase 2 subunit alpha (CSNK2A2), CTNNB1, HDAC1 and PPP2R1B, as well as an increase in peptidyl-prolyl cis–trans isomerase NIMA-interacting 1 (PIN1) (see Supplementary Figure S1B). WNT/β-catenin signaling is a key regulator of cellular metabolism, including cell proliferation, tissue maintenance and remodeling [102]. However, it has been shown that oxidative stress is able to inhibit histone deacetylases (HDAC) [103,104] and serine/threonine phosphatase 2A proteins like PPP2R1B [73], which could stimulate the activation of WNT/β-catenin signaling [105,106]. Moreover, PIN1 has been shown to be a positive regulator of WNT/β-catenin signaling [107]. Interestingly, Martowicz et al. (2019) reported that the activation of WNT/β-catenin signaling is involved in retinal angiogenesis [108], which could lead to glaucoma [109]. In addition, some studies have shown that the overexpression of WNT/β-catenin signaling could exacerbate the pathological features of retinal diseases such as AMD and DR through the induction of retinal inflammation and oxidative stress [110,111].

## 5. Conclusions

In conclusion, the findings of this study showed that glutamate- and hydrogen peroxide (H_2_O_2_)-induced toxicity in R28 cells could exhibit similar pathological events to those observed in glaucoma. This may provide a solid basis for using the neuroretinal R28 cells as a tool to develop new therapeutic approaches for oxidative stress-induced retinal diseases.

## Data Availability

The data presented in this study are available in the published article and its Supplementary Materials.

## Supplementary Materials

The following supporting information can be downloaded at https://www.mdpi.com/article/10.3390/cells13090775/s1: Supplementary File S1: Output data resulting from the MaxQuant analysis of the MS raw data; Supplementary File S2: MS identification of proteins in the glutamate-exposed cells compared to the control and the statistical analysis of significantly altered proteins in both groups; Supplementary File S3: MS identification of proteins in the cells exposed to H_2_O_2_ compared to the control and the statistical analysis of significantly differentially expressed proteins in the two groups; Supplementary Figure S1: Differential expression of significantly changed proteins involved in the most affected canonical signaling pathways after the exposure of R28 cells to glutamate and H_2_O_2_ (FDR < 1%; *p* < 0.05).
