# Supplementary figures and images for: Neuroretinal Cell Culture Model as a Tool for the Development of New Therapeutic Approaches for Oxidative Stress-Induced Ocular Diseases, with a Focus on Glaucoma
Source: Cells. 2024 May 1;13(9):775. doi: 10.3390/cells13090775 (PMC11083839; doi:10.3390/cells13090775)

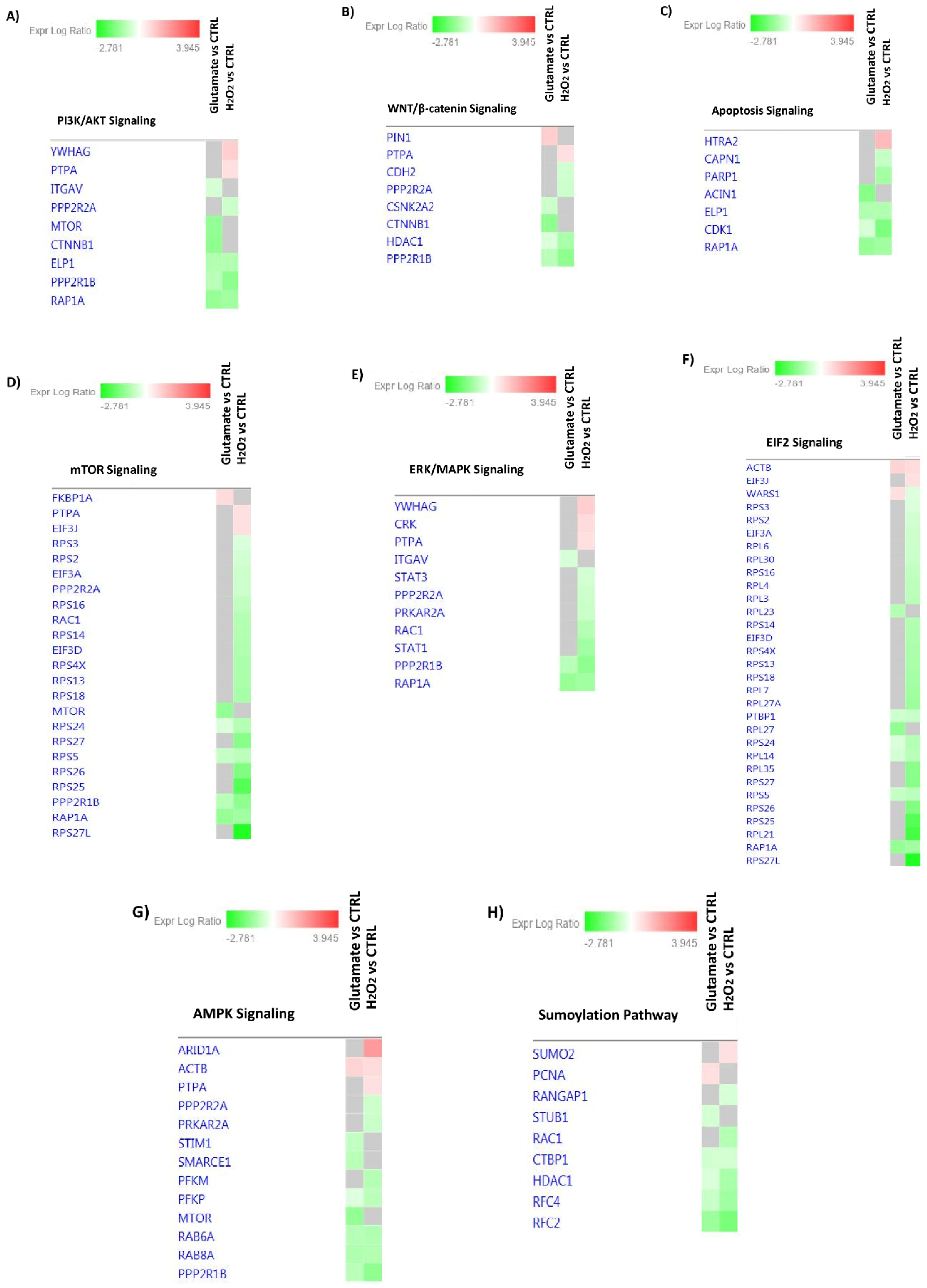

Supplement: Supplementary file 1 [file cells-13-00775-s001.zip › Supplementary Figure S1.tif]
